# Development and Validation of a Self-Report Measure of Mentalizing: The Reflective Functioning Questionnaire

**DOI:** 10.1371/journal.pone.0158678

**Published:** 2016-07-08

**Authors:** Peter Fonagy, Patrick Luyten, Alesia Moulton-Perkins, Ya-Wen Lee, Fiona Warren, Susan Howard, Rosanna Ghinai, Pasco Fearon, Benedicte Lowyck

**Affiliations:** 1 Research Department of Clinical, Educational and Health Psychology, UCL, London, United Kingdom; 2 Faculty of Psychology and Educational Sciences, KU Leuven, Leuven, Belgium; 3 Education and Training Department, Sussex Partnership NHS Foundation Trust, Worthing, United Kingdom; 4 Freelance Researcher and Trainer, Guildford, United Kingdom; 5 Department of Psychology, University of Surrey, Guildford, United Kingdom; 6 Faculty of Medicine, KU Leuven, Leuven, Belgium; University of Hertfordshire, UNITED KINGDOM

## Abstract

Reflective functioning or mentalizing is the capacity to interpret both the self and others in terms of internal mental states such as feelings, wishes, goals, desires, and attitudes. This paper is part of a series of papers outlining the development and psychometric features of a new self-report measure, the Reflective Functioning Questionnaire (RFQ), designed to provide an easy to administer self-report measure of mentalizing. We describe the development and initial validation of the RFQ in three studies. Study 1 focuses on the development of the RFQ, its factor structure and construct validity in a sample of patients with Borderline Personality Disorder (BPD) and Eating Disorder (ED) (n = 108) and normal controls (n = 295). Study 2 aims to replicate these findings in a fresh sample of 129 patients with personality disorder and 281 normal controls. Study 3 addresses the relationship between the RFQ, parental reflective functioning and infant attachment status as assessed with the Strange Situation Procedure (SSP) in a sample of 136 community mothers and their infants. In both Study 1 and 2, confirmatory factor analyses yielded two factors assessing Certainty (RFQ_C) and Uncertainty (RFQ_U) about the mental states of self and others. These two factors were relatively distinct, invariant across clinical and non-clinical samples, had satisfactory internal consistency and test–retest stability, and were largely unrelated to demographic features. The scales discriminated between patients and controls, and were significantly and in theoretically predicted ways correlated with measures of empathy, mindfulness and perspective-taking, and with both self-reported and clinician-reported measures of borderline personality features and other indices of maladaptive personality functioning. Furthermore, the RFQ scales were associated with levels of parental reflective functioning, which in turn predicted infant attachment status in the SSP. Overall, this study lends preliminary support for the RFQ as a screening measure of reflective functioning. Further research is needed, however, to investigate in more detail the psychometric qualities of the RFQ.

## Introduction

The term reflective functioning (RF) (here used synonymously with the term mentalizing) was first popularized through work on borderline personality disorder (BPD) [1–4] and parent–infant attachment [5, 6]. The notion of mentalizing refers to the capacity to reflect on internal mental states such as feelings, wishes, goals, and attitudes, with regard to both the self and others. Studies suggest that this capacity develops in the context of secure attachment relationships. By contrast, disruptions in attachment relationships, most likely in interaction with environmental and genetic vulnerability, have been associated with impairments in mentalizing [6, 7]. Such impairments have been shown to play a key role in a variety of disorders and problem behaviors such as BPD [8], eating disorders (EDs) [9, 10], depression [11], and antisocial personality disorder [12]. These ideas have also inspired a number of mentalization-focused interventions that have received some empirical support in both randomized controlled trials and naturalistic studies [8, 13–18].

Although several self-report measures have been developed to assess constructs related to mentalizing [19, 20], such as mindfulness, perspective-taking, empathy, theory of mind, alexithymia, and psychological mindedness, no self-report questionnaire of RF currently exist [20]. The only currently well-validated measures that directly assess RF are both interview-based: the Reflective Functioning Scale (RFS) [21] applied to the Adult Attachment Interview (AAI) [22] and the Parent Development Interview [23] applied to an interview about parenting. However, because it is time- and labour-intensive, and requires highly trained raters, sample sizes tend to be small [24]. There is a need for an instrument suitable for use in large-scale epidemiological studies where hypotheses about the significance of failures in mentalizing in personality disorder, trauma, and environments associated with insecure patterns of attachment could be examined. For this purpose, a generic self-report measure of RF for adults is urgently needed.

### The present study

This paper is the first in a series outlining the psychometric properties of a new self-report measure of mentalization, the Reflective Functioning Questionnaire (RFQ). This paper describes the development and initial validation of this measure in three studies. Here, we describe the general aims of these studies. The rationale, design, and hypotheses of each of the studies are outlined in more detail in each section. Because mentalization-based approaches concerning personality disorders and EDs are most extensively developed, and the disorders often co-occur [25–27], we focused on these disorders first. Study 1 focused on the development of the RFQ and its factor structure in a sample of patients with BPD and EDs (n = 108) and a sample of normal controls (n = 295). This study also investigated the construct validity of the RFQ by investigating its discriminatory, convergent, and divergent validity. Study 2 investigated the factor structure and construct validity of the RFQ in a sample of 129 carefully screened patients with personality disorder and 281 normal controls. Study 3 addressed one of the key predictions of mentalizing approaches—that is, that RF is associated with parental RF and infant attachment status as assessed in the Strange Situation Procedure—in a sample of 136 community mothers and their infants.

## Study 1: Development and preliminary validation of the RFQ

### Development of the RFQ

In developing a self-report measure of mentalizing researchers face a major problem. The very capacity that we aim to assess is needed to complete a measure of the capacity: individuals need to rely on their capacity for mentalizing in responding to questions about mentalizing. How can anyone self-reflect accurately and arrive at the conclusion that they are poor at self-reflection? For instance, when asked “I don’t always know why I do what I do”, individuals have to be able to take a meta-perspective with regard to their own mental states, that is, they have to mentalize. Limitations in self-knowledge and consequent biases associated with assessment of personality features through self-report questionnaires are well demonstrated [28]. Research confirms that assessments of RF and social cognition more generally are particularly vulnerable to this limitation [29]. Indeed, RF occurs largely outside conscious awareness or conscious control, and the individual may have little or no privileged access to their ability to function in this domain [20, 30]. Of course, the response of others should yield reliable corrective information in relation to challenges an individual faces in mentalizing. Yet, the absence of a capacity to reflect accurately on the experiences of others may deprive that individual from arriving at an accurate interpretation of that corrective social experience. Thus, we expect individuals to be biased with regard to their own capacity for RF, and those with limited reflective ability may commonly be unaware that they experience mentalizing difficulties. Simple questions such as “I have no difficulty in understanding others’ motives” are unlikely to discriminate between poor and good mentalizing.

Two broad types of impairments in RF have been described and have been shown to be implicated in vulnerability for psychopathology [25, 31]. The first impairment involves what is called *hypomentalizing*, or concrete or psychic equivalent thinking, reflecting an inability to consider complex models of one’s own mind and/or that of others. Hypomentalizing has been related to vulnerability for a wide range of disorders, including BPD [25], EDs [9], and depression [32, 33]. Although individuals who are prone to hypomentalizing may be aware of their limitations in their understanding of themselves and/or others, this is not necessarily the case [34]. For instance, BPD patients often score normally on self-report questionnaire measures of empathy [35], a core component of RF with regard to others, while they typically perform worse than normal controls on experimental tasks assessing cognitive empathy [34, 36]. Thus, hypomentalizing may compromise accurate responding to questionnaires.

Individuals who show the opposite tendency, *hypermentalizing*, also termed pseudomentalizing or excessive mentalizing [34], may introduce a different kind of bias into their self-report. Hypermentalizing is the generation of mentalistic representations of actions without appropriate evidence available to support these models. The tendency to develop inaccurate models of the mind of oneself and others is typically reflected in long and overly detailed accounts that have little or no relationship to observable (testable) reality. Furthermore, individuals who are prone to hypermentalizing may experience themselves as particularly good mentalizers based on the sheer volume of their mentalizing output, and will therefore also show biased responses to self-report measures of RF.

By contrast, genuine mentalizing is characterized by a recognition of the opaqueness of mental states [31, 37]. Hence, a genuine mentalizing stance is characterized by modesty about knowing one’s own mental states and humility in relation to knowing the mental states of others. Both occur in combination with an observed ability to form relatively accurate models of the mind of self and others. That is, individuals with high RF can be expected to demonstrate some certainty about their own mental states and those of others, while at the same being aware that their certainty should be conditioned by knowledge that mental states are ultimately opaque. By contrast, for individuals who show a tendency for hypermentalizing, endorsing items assessing RF may reflect attempts to defensively bolster their self-esteem (e.g., “*Of course I always know why I do what I do*”) despite their objectively observed difficulties in providing plausible accounts of the putative motives of their actions.

Consideration of the complexity of the relationship of self-report and mentalizing ability suggests that a simple continuous scoring system to assess RF might conflate these two types of impairments in RF. Stated otherwise, simple polar-scored items assessing RF (i.e., with higher scores assumed to assess higher levels of mentalizing) may not accurately capture an individual’s capacity for mentalizing. Indeed, from a mentalizing perspective, extreme responses on both ends of a Likert-type scale may indicate qualitatively different impairments in mentalizing. For example, someone who strongly agrees with a statement like “*I always know what I feel*” may be too certain about his/her mental states, reflecting mentalizing beyond the evidence (hypermentalizing) [31]. On the other hand, individuals who strongly disagree with this item may have very little understanding of their own inner mental states, reflecting hypomentalizing. One could of course rescore such items so that middle scores reflect the most adaptive scores, but this would again lead to conflating two potentially very different impairments in RF (i.e., hypomentalizing and hypermentalizing). The distinction is all the more relevant as both types of impairments in RF might be differentially related to different types, or different aspects, of psychopathology [34, 38]. While individuals with BPD may show both hypomentalizing and occasional hypermentalizing, depending on the emotional context, individuals with anorexia nervosa often show marked hypermentalizing or hyper-reflectivity [39]. Also, trajectories of change as a result of maturation or psychosocial interventions might differ for both types of impairments, and they might have different developmental and neurobiological underpinnings [25, 40, 41].

In developing the RFQ, we first developed a set of items that were scored using a polar-scoring and median-scoring method (for further details, see below). For *polar-scored* items, stronger agreement (or disagreement in case of inverted items) yielded higher RF scores (e.g., “*I realize that I can sometimes misunderstand my best friends*” or “*I get confused when people talk about their feelings*”). *Median-scored* items were designed so that responses reflecting an awareness of the opaqueness of mental states (“disagree somewhat” or “agree somewhat”) received the highest scores, while extreme answers (“strongly agree” or “strongly disagree”) were scored so that they reflected lower scores. For example, the response to the item “*I always know what I feel*”, which was scored on a 6-point Likert-type scale ranging from 1 (completely disagree) to 6 (complete agree), was rescored as 1, 2, 3, 3, 2, 1, so that the more extreme the rating, the lower the score on RF. Extensive analyses of both scoring systems using Principal Component Analysis (PCA) and Confirmatory Factor Analysis (CFA) in both community and clinical samples failed to yield evidence for the construct validity of either polar- or median-scored scales. We therefore decided to recode all items to be congruent with the theoretical assumptions summarized above. We thus developed a scale assessing *Certainty about Mental States* (RFQ_C), which focused on the extent to which individuals disagree with statements such as “*I don’t always know why I do what I do*”, rescoring these items so that low agreement on this scale reflected hypermentalizing, while high agreement reflected more genuine mentalizing (acknowledging the opaqueness of mental states). Initially, we used a 6-point Likert-type scale so that items were rescored to 2, 1, 0, 0, 0, 0. To increase the range of scores, after initial pilot studies, we changed the scale to a 7-point Likert-type scale rescoring these items to 3, 2, 1, 0, 0, 0, 0 [42]. Similarly, we developed a scale assessing *Uncertainty about mental states* (RFQ_U), which in the extreme was expected to assess hypomentalizing. Responses to items such as “*Sometimes I do things without really knowing why*” were recoded to 0, 0, 0, 0, 1, 2 (6-point Likert-type scale) or 0, 0, 0, 0, 1, 2, 3 (7-point Likert-type scale), so that very high scores reflected a stance characterized by an almost complete lack of knowledge about mental states, while lower scores reflected acknowledgment of the opaqueness of one’s own mental states and that of others, characteristic of genuine mentalizing. As our aim was to develop a brief screening measure of RF, we selected the six items that had the highest loading on their respective factor across a series of exploratory and confirmatory factor analyses across the samples used in this paper.

### Study hypotheses

We investigated the reliability and validity of the RFQ_C and RFQ_U subscales in a sample of normal controls (n = 295) and BPD patients with comorbid EDs (n = 108):

First, exploratory factor analysis (EFA) and (multi-group) confirmatory factor analysis (CFA) was used to investigate the factor structure and factor invariance across the patient and control samples. Congruent with theoretical formulations, we expected a two-factor model, with scales assessing certainty and uncertainty about mental states of self and others, to provide the best fit to the data in both samples.

Next, we investigated the internal consistency and test–retest reliability of these two subscales, as well as their relationships with demographic factors.

The discriminant validity of the RFQ was investigated by testing its ability to differentiate between participants in the clinical and non-clinical samples. We also computed correlations between the RFQ scales and core features of psychopathology typical of borderline patients and eating disordered patients, that is, severity of depression, both self-report and clinician-rated BPD and ED features, as well as impulsivity.

The convergent and divergent validity of the RFQ was measured in the non-clinical sample by relating the RFQ to measures assessing concepts that have been closely related to RF [19, 20]. We expected the RFQ subscales to be positively correlated with other measures of internally based mentalizing, such as mindfulness as assessed with the Mindful Awareness Attention Scale, perspective-taking as assessed by the Perspective Taking Subscale of the Interpersonal Reactivity Index [43], and empathy as assessed by the Basic Empathy Scale. We also expected the RFQ scales to be significantly positively related to externally based mentalizing as assessed with the Reading the Mind in the Eyes Test, although these correlations were expected to be lower given that internally focused and externally based mentalizing are two relatively distinct capacities [20].

### Methods

#### Scale development

In the initial development of the scale, 101 statements were constructed so that level of agreement corresponded to high or low RF. Responses were rated across a 6-point Likert scale ranging from “strongly disagree” (= 1) to “strongly agree” (= 6). High RF was indicated on polar response items by either strong agreement (= 6) or strong disagreement (= 1) with the statement. For example, a high mentalizing participant would strongly agree (= 6) with the statement “*I’m often curious about the meaning behind others’ actions*” because it assesses the degree of motivation to reflect about intentional states in others. On the other hand, a high mentalizer would strongly disagree (= 1) with the statement “*I frequently feel that my mind is empty*”. We also included central response items, with disagreeing somewhat (= 3) or agreeing somewhat (= 4) indicating high RF. These central scoring items were constructed in an attempt to elicit a balanced mentalizing perspective, whereby the participant must recognize that they can know something, but not everything about a person. For example, “*I can tell how someone is feeling by looking in their eyes*” assesses the degree to which the respondent is realistic about their ability to mind-read another’s body language. Hence, these items are scored as deviations from the midpoint, with a high mentalizing response rated as disagree somewhat (= 3) or agree somewhat (= 4).

These 101 items were rated by 14 international experts, who indicated whether they thought each statement reflected mentalizing skills or not. After rejecting 31 insufficiently reliable items, 70 remaining items were re-examined by the same 14 experts for face and content validity. Badly worded, repetitious, or irrelevant items were rejected. After this process, 46 items were retained. As noted, initial exploratory analyses demonstrated that the polar-scored items were prone to bias in assessing RF, because they conflated hypomentalizing and hypermentalizing (for example, high scores on these polar scores were negatively related with measures of mindfulness). We therefore decided to focus on the 26 central response items, and recoded these to assess Certainty about Mental States (RFQ_C) and Uncertainty about Mental States (RFQ_U). For the RFQ_C subscales, items were rescored to 2, 1, 0, 0, 0, 0. For the RFQ_U scale, responses were recoded to 0, 0, 0, 0, 1, 2. In summary, the RFQ_C and RFQ_U scales were scored by recoding the same 26 items.

#### Participants and procedures

The RFQ was administered to 295 non-clinical controls (83 students and non-academic staff from two colleges, and 212 university staff) after obtaining written informed consent. Participants were recruited by researchers approaching participants directly, or indirectly, whereby questionnaire packs were distributed in central locations (e.g., dining areas) for participants to pick up themselves. Completed questionnaire packs were placed in secure central collection points.

The clinical sample consisted of outpatients with BPD and ED who completed a battery of measures after obtaining written informed consent at assessment interview, and included 53 patients from three personality disorder clinical units and 55 outpatients from two ED units in the United Kingdom. Participants in the personality disorder sample were recruited from one National Health Service (NHS) specialist outpatient service and two service-user-led services. Participants in ED samples were outpatients from two specialist NHS clinician-led services; all had DSM-IV ED diagnoses. Twenty-four percent of patients in the two ED services also met diagnostic criteria for BPD on the SCID-II, and on the clinician-rated Zanarini scale [44], a continuous measure of BPD symptoms, comorbidity reached 38%.

Fifty participants (30 non-clinical controls and 20 ED and BPD outpatients) repeated the RFQ approximately 3 weeks after initial administration to establish test–retest reliability.

This study was approved by University College London Research Ethics Committee, by the University of Surrey Faculty of Arts and Human Sciences Ethics Committee, and by the NHS National Research Ethics Service.

#### Measures

**Mindfulness:** The 15-item self-report Mindful Awareness Attention Scale (MAAS) [45] has been shown to positively correlate with measures of emotional intelligence and mental well-being and has good internal reliability (α = 0.82). It is used to measure participants’ ability to attend to and be fully aware of present-moment experience without acting on “autopilot” or being preoccupied. Internal reliability in the present non-clinical sample was excellent (α = 0.85).

**Empathy:** The cognitive subscale of the Basic Empathy Scale (BES) [46], a 9-item measure with good psychometric properties (Cronbach’s α = 0.79) was used to assess empathy. Internal reliability in the present non-clinical (α = 0.76) and clinical samples (α = 0.79) was good. Internal reliability in the present non-clinical sample was reasonable (α = 0.65).

**Perspective-taking:** The 7-item Perspective-Taking Subscale (PTS) of the Interpersonal Reactivity Index [43] was used to assess perspective-taking capacities (α = 0.67).

Participants in both samples also completed measures of disordered eating, impulsivity, and severity of depression. Disordered eating was assessed with the 26-item self-report Eating Attitudes Test (EAT) [47], which generates an overall score of disordered eating attitudes, as well as three subscales: dieting, bulimia and oral control. Reliability for the EAT is high. In addition, the authors recommend using a cut-off score of ≥20 to identify participants with a likely diagnosis of ED. Internal reliability in the present non-clinical sample (α = 0.83) and clinical sample (α = 0.94) was excellent.

Impulsivity was assessed using the 22-item self-report Multi-Impulsivity Scale (MIS) [48]. The MIS measures 11 impulses and behaviors, including food and non-food items. In the present study the “Do” subscale was applied as the main measure of impulsivity. Internal reliability was low in the present non-clinical sample (α = 0.49) but much higher in the clinical sample (α = 0.68).

Severity of depression was measured using the 21-item self-report Beck Depression Inventory-II (BDI) [49]. Internal reliability in the present non-clinical (α = 0.91) and non-clinical (α = 0.94) samples was excellent.

**Borderline features:** Participants in both samples completed the Borderline Personality Inventory (BPI) [50], a 51-item true/false self-report inventory that is based on Kernberg’s model of borderline personality organization [51] but is also compatible with DSM criteria. It demonstrates strong internal consistency (α = 0.91) and has good sensitivity and specificity for identifying BPD caseness. The authors suggest a cut-off score of 10 on the 20 most discriminatory items as most likely to identify a DSM diagnosis of BPD. An overall continuous measure of BPD was also generated with higher scores indicating more borderline features. Internal reliability in the present non-clinical (α = 0.89) and clinical samples (α = 0.94) was excellent.

In the clinical sample, the Zanarini Rating Scale for Borderline Personality Disorder (ZAN) [44] was also administered. The ZAN is a clinician-rated interview that has been shown to reliably predict BPD diagnostic status as well as being sensitive to change. In the present study a subsample of clinical participants were administered the ZAN by their treating psychiatrist as part of their assessment interview. Questions are measured on a 5-point anchored rating scale from 0–4, yielding a total score of 0–36. Ratings represent both frequency and severity of symptoms where 0 = no symptoms, 1 = mild symptoms, 2 = moderate symptoms, 3 = serious symptoms, and 4 = severe symptoms. A total scale score and four subscales are derived from the 9 questions: affective disturbance, cognitive disturbance, impulsivity and disturbed relationships. For the purposes of the present study, a diagnostic categorical variable was created by applying a cut-off score of 10. Participants with scores exceeding this were deemed likely to have a DSM diagnosis of BPD. This translated as scoring at least “moderately” distressed on 5 of the 9 questions, each one representing DSM criteria for BPD. Internal reliability of the ZAN Total Score in the present clinical sample was excellent (α = 0.86)

For clinical participants, clinicians were also provided with a record sheet where they were required to check off symptoms from DSM diagnostic criteria for BPD, anorexia nervosa, bulimia nervosa, and eating disorder not otherwise specified [52]. This generated a score from 0 to 9, where 0 = no criteria met and 9 = all nine criteria met.

#### Statistical analyses

The factor structure of the RFQ was first investigated using (multi-group) CFA with maximum likelihood estimation using the maximum likelihood method in AMOS (Version 4.01) [53]. In order to evaluate the goodness of fit of the factor structure, the following fit indices were used: the χ^2^/df ratio, the root mean square error of approximation (RMSEA) and two-sided 90% confidence intervals, the comparative fit index (CFI), and the non-normed fit index (NNFI). A model in which χ^2^/df was ≤3, the CFI and NNFI values were greater than 0.90, and the RMSEA index was between 0.00 and 0.06 with confidence intervals between 0.00 and 0.08 [54] was considered acceptable. Consistent with state-of-the-art recommendations [55, 56], we limited the number of possible error correlations to a minimum, allowing only error correlations between items that were similar in formulation or meaning (e.g., item 17, “I don’t always know why I do what I do” and item 36, “Sometimes I do things without really knowing why”.). Multi-group CFA with maximum likelihood estimation was used to investigate the factorial invariance of the RFQ in patients and controls, following state of the art recommendations [57–59]. This multi-group comparison compared a fully unconstrained model (Model 1); a measurement weights model (Model 2) fixing the factor loadings; a measurement intercepts model (Model 3), which fixed the factor loadings and intercepts; a structural covariances model (Model 4), which also fixed the variance of the factors across patients and controls; and a measurement residuals model (Model 5), which also fixed the covariances and variances of the errors. In order to compare these five models, *χ*^*2*^-difference tests were used.

Correlational analyses on RFQ Time 1 and Time 2 data were used to establish test–retest reliability. Simple *t*-tests and binary regression analyses were used to investigate the ability of the RFQ to discriminate between BPD and ED patients and normal controls. All odds ratios (ORs) are expressed as absolute values. The convergent and divergent validity of the RFQ was assessed by computing Pearson correlations between the RFQ subscales and demographic variables and related constructs.

### Results

#### Demographics

The overall sample was predominantly female, a trend more pronounced in the clinical than the non-clinical sample (81%, 61%) (Table 1). This difference was significant (χ^2^(1) = 14.62, *p*<0.001), although the strength of association was small (*Φ* = 0.19). Non-clinical participants generally had higher socioeconomic status than clinical participants, with more non-clinical than clinical participants employed in management and professional occupations (χ^2^(2) = 14.98, *p* = 0.001), a medium effect (Cramer’s *V* = 0.20). The non-clinical group (*M* = 32.68, *SD* = 13.14) was significantly younger than the clinical group (*M* = 36.08, *SD* = 11.52; *t*(204.09) = 2.49, *p* = 0.01). Differences between number of people in a stable relationship or married versus those who were not did not differ significantly between clinical and non-clinical (χ^2^(1) = 0.02, *p* = 0.88) groups. Most participants were of White ethnic origin (92%). Years in education was similar between clinical (*M* = 15.49, *SD* = 3.93) and non-clinical (*M* = 15.47, *SD* = 2.92) groups.

**Table 1:** Demographic characteristics of participants in the patient and control samples.

	Clinical		Non-clinical	
	*M*	*SD*	*M*	*SD*
**Age**	36.08	11.52	32.68	13.14
**Years in Education**	15.49	3.93	15.47	2.92
	*N*	%	*N*	%
**Gender**				
Male	19	19	116	39
Female	83	81	178	61
**Relationship**				
Long-term relationship	36	37	103	36
No long-term relationship	62	63	184	64
**Occupation**				
Managerial and professional occupations	27	33	154	55
Intermediate occupations	27	33	79	28
Routine and manual occupations	28	34	49	17
**Employment status**				
Employed/Student	51	49	79	95
Unemployed/Unclassifiable	53	51	4	5
**Ethnicity**				
White (British/Irish/Any other White)	76	93	59	92
Non-White (Mixed, Black, Asian, Chinese)	6	7	5	8

#### Confirmatory factor analyses

**Fig 1:**
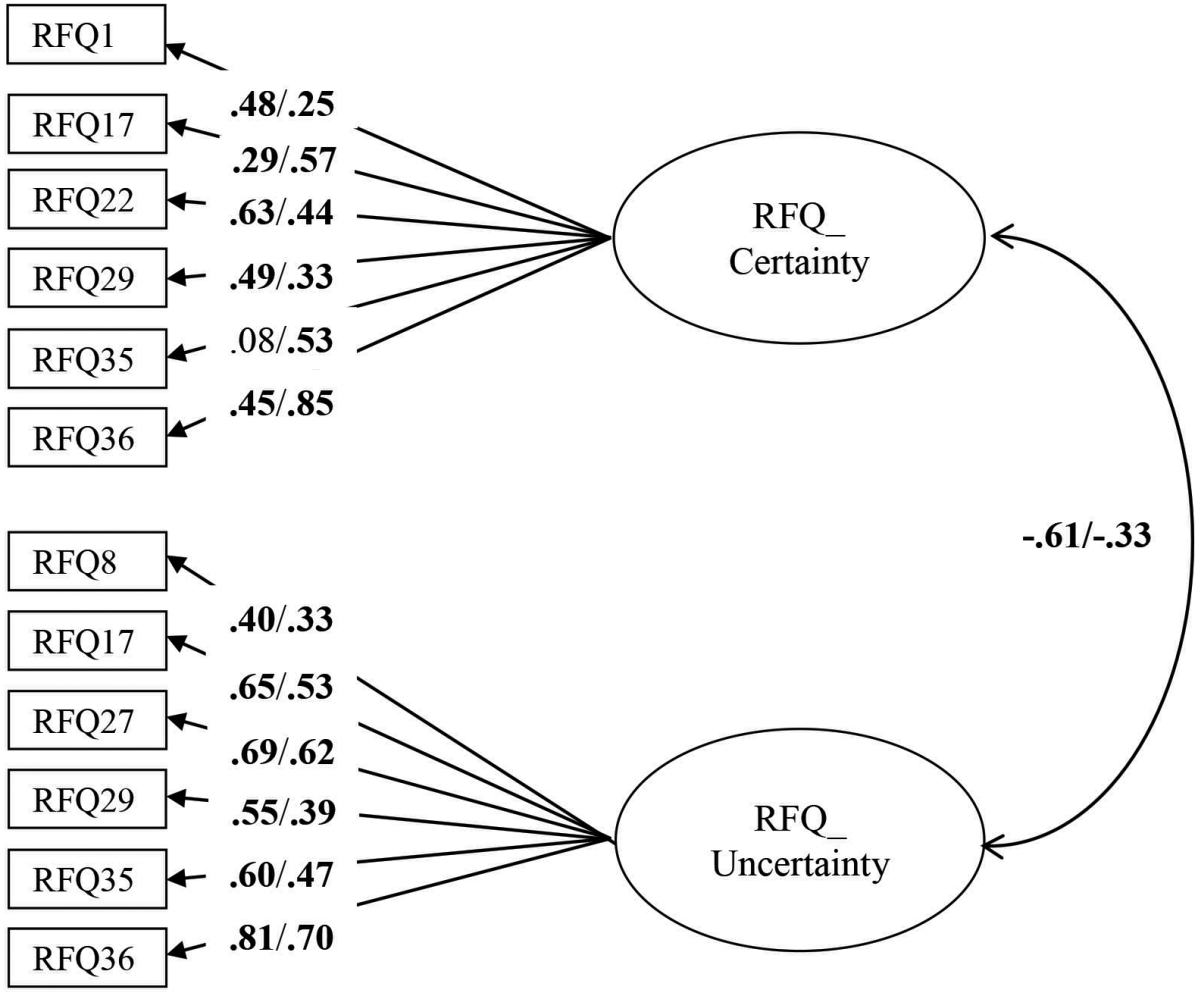
Multi-group CFA with factor loadings in the clinical (left) and the non-clinical (right) sample, respectively. Residuals and correlations between residuals are omitted for clarity of presentation. Rectangles indicate measured variables and circles represent latent constructs. Standardized maximum likelihood parameters are used. Bold estimates are statistically significant at *p*<0.05 (two-tailed).

The RFQ_C and RFQ_U items were subjected to a CFA using data from the clinical and non-clinical sample combined. The initial model did not provide a good fit to the data, χ^2^/df = 6.03; RMSEA = 0.11 (95% confidence interval [CI] = 0.10–0.13); CFI = 0.78, NNFI = 0.71. Modification indices suggested adding error covariances between several items with similar item content and/or wording, which resulted in a model with a good fit: χ^2^/df = 2.2; RMSEA = 0.06 (CI = 0.04–0.07); CFI = 0.95, NNFI = 0.93. All items had substantial and significant loadings in the expected direction on their respective factors. Next, a multi-group CFA tested the invariance of this model across both groups. The fully unconstrained model had a good fit, χ^2^/df = 1.82; RMSEA = 0.05 (CI = 0.03–0.06); CFI = 0.92, NNFI = 0.90), suggesting factorial invariance across both samples. All subsequent models led to a significantly worse fit (measurement weights model: Δχ^2^ = 60.76, *p*<0.01; measurement intercepts model Δχ^2^ = 185.12, *p*<0.001; structural covariances model Δχ^2^ = 185.25, *p*<0.001; measurement residuals model Δχ^2^ = 322.64, *p*<0.001), suggesting that an unconstrained model fitted the data best. As can be seen in Fig 1, for some items, item loadings differed between the clinical and non-clinical sample. In addition, the estimated correlation between the RFQ_C and RFC_U subscales was higher in the clinical sample compared with the non-clinical sample. The size of these correlations suggests that these two subscales are relatively independent, particularly in the non-clinical sample (with 37% and 11% of the variance shared in the non-clinical and clinical sample, respectively).

Estimates of internal consistency for RFQ_U and RFQ_C were 0.77 and 0.65 in the clinical sample, and 0.63 and 0.67 in the non-clinical sample. The test–retest reliability over a period of 3 weeks was excellent, with *r*s = 0.84 and 0.75 for RFQ_U and RFQ_C, respectively, all *p*s<0.001.

#### Correlations with demographic features

Both RFQ subscales were unrelated to demographic features in both the clinical and non-clinical samples, with two small exceptions. RFQ_U was slightly but significantly negatively correlated with age (*r* = -0.13, *p* = 0.003) in the non-clinical sample, suggesting that uncertainty about mental states might slightly decrease with age. Furthermore, in the non-clinical sample, participants who were currently in a romantic relationship had lower scores on the RFQ_U (M = 1.15, SD = 1.82) compared with participants who were not in a romantic relationship (M = 1.96, SD = 2.01, *t* = -2.15, *p =* 0.003), which might suggest that strong attachment relationships might reduce uncertainty about subjective experiences.

#### Group differences

The RFQ_C (*t* = -0.209, *p*<0.04) and particularly the RFQ_U (*t* = 8.99, p<0.001) differentiated between clinical and non-clinical participants (Table 2). Logistic regression analyses confirmed that the RFQ_U subscale was superior in discriminating between the two samples (Table 3). Whereas the RFQ_C scale showed only a trend towards predicting the odds of belonging to the nonclinical sample (OR = 1.06, CI = 0.96–1.27, *p* = 0.19), the RFQ_U was highly significantly associated with a higher odds of belonging to the clinical sample (OR = 1.60, CI = 1.42–1.79, *p*<0.001).

**Table 2:** Means and standard deviations for the RFQ subscales.

	Clinical or non-clinical group	n	Mean	SD
RFQ_C	Clinical	103	1.48	1.98
	Non-clinical	291	1.98	2.16
RFQ_U	Clinical	103	4.91	3.36
	Non-clinical	291	1.77	1.95

**Table 3:** Association between the RFQ subscales and clinical features.

	Odds Ratio	CI
Clinical versus non-clinical		
RFQ_C	1.06	0.96–1.27
RFQ_U	1.60***	1.42–1.79
BPD diagnosis (Zanarini)		
RFQ_C	1.53***	1.10–2.11
RFQ_U	1.29**	1.08–1.54
BDP diagnosis by clinicians		
RFQ_C	1.76	0.99–2.52
RFQ_U	1.20*	1.06–1.44
BPD diagnosis BPI cut-off		
RFQ_C	1.40*	1.02–1.92
RFQ_U	1.35***	1.12–1.64
BPD–eating disorder comorbidity		
RFQ_C	1.96**	1.10–3.50
RFQ_U	1.05	0.87–1.26
Self-harm		
RFQ_C	.98	0.65–1.47
RFQ_U	1.41***	1.15–1.73

Note:

*p<0.05,

**p<0.01,

***p<0.001

With regard to interview-based measures in the clinical sample, both RFQ_U and RFQ_C were associated with BPD diagnosis as assessed with the ZAN (Table 2). Higher scores for the RFQ_U (OR = 1.29, CI = 1.08–1.54, *p* = 0.005) and lower scores for the RF_C (OR = 1.53, CI = 1.10–2.11, *p* = 0.01) were associated with BPD caseness. For clinician-reported diagnosis of BPD, the RFQ_C scale marginally (OR = 1.76, CI = 0.99–2.52, *p* = 0.06) and the RFQ_U scale (OR = 1.20, CI = 1.06–1.44, *p* = 0.04) significantly differentiated those with the diagnosis. In both the clinical and non-clinical sample, RFQ_U was significantly associated with BPD diagnosis based on the BPI cut-off, with higher scores predicting caseness (OR = 1.35, CI = 1.12–1.64, *p* = 0.001 in the clinical sample, and OR = 1.51, CI = 1.04–2.19, *p* = 0.03 in the non-clinical sample). The RFQ_C scale was associated with BPD caseness based on the BPI only in the clinical sample (OR = 1.40, CI = 1.02–1.92, *p* = 0.04 in the clinical sample, and OR = 1.06, CI = 0.66–1.76, *p* = 0.80 in the non-clinical sample), with lower scores being associated with caseness.

However, the RFQ_C scale was significantly associated with comorbidity between BPD and ED diagnosis (OR = 1.96, CI = 1.10–3.50, *p* = 0.002), whereas the RFQ_U was not (OR = 1.05, CI = 0.87–1.26, *p* = 0.64). Finally, RFQ_U was also highly significantly associated with clinician-reported self-harm (OR = 1.41, CI = 1.15–1.73, *p* = 0.001), whereas the RFQ_C was not (OR = 0.98, CI = 0.65–1.47, *p* = 0.90).

#### Correlations with clinical features

Correlations with clinical features obtained from self-report measures are shown in Table 4. The RFQ_U was, congruent with expectations, highly positively correlated in both the clinical and non-clinical samples with borderline features as assessed with the BPI, with severity of depression as measured with the BDI, and with impulsivity as assessed with the Do Subscale. In the clinical sample, but not in the non-clinical sample, the RFQ_U was also associated with severity of ED features as assessed with the EAT.

**Table 4:** Relationships between the RFQ subscales and clinical features.

	BPI	EAT	BDI	Impulsivity
RFQ_C	-0.47**/-0.33**	0.08/-0.01	-0.26*/-0.24**	-0.28*/-0.30**
RFQ_U	0.53**/0.41**	0.22*/0.13	0.53**/0.40**	0.45**/0.38**

Note 1:

*p<0.05,

**p<0.01,

***p<0.001

Note 2: Correlations are for clinical/non-clinical sample, respectively.

The RFQ_C was negatively correlated with borderline features as assessed with the BPI, severity of depression and impulsivity, but was not correlated with the EAT.

#### Correlations with related constructs

Correlations with related measures are shown in Table 5. The RFQ_C was, as expected, positively related to other measures of internally based mentalizing, such as the BES and mindfulness as assessed with the MAAS, but was, against expectations, not related to perspective-taking. The RFQ_U was, against expectations, not related to empathy, but was negatively related to mindfulness and perspective-taking.

**Table 5:** Relationships between the RFQ subscales and related constructs.

	BES	MAAS	Perspective-taking	RMET
RFQ_C	0.30**/0.35**	-/0.34**	-/0.06	0.14/0.18*
RFQ_U	-0.05/-0.07	-/-0.33**	-/-0.18*	0.03/-0.17*

Note 1:

*p<0.05,

**p<0.01,

***p<0.001

Note 2: Correlations are for clinical/non-clinical sample respectively.

The RFQ_C was slightly positively correlated with the RMET as a measure of externally based mentalizing, but this association was significant only in the non-clinical sample (r = 0.18, *p*<0.05). The RFQ_U was slightly negatively related to externally based mentalizing as assessed with the RMET, but only in the non-clinical sample (r = -0.17, *p*<0.05).

### Discussion and conclusions

The results of Study 1 provide preliminary support for the factorial invariance of the RFQ in a clinical sample and a non-clinical sample, as well as for the internal consistency and test–retest reliability. CFA suggested a two-factor structure that was invariant across the clinical and non-clinical samples, although an unconstrained model fitted the data best, suggesting, as might be expected, differences in the measurement weights, measurement intercepts, structural covariances, and measurement residuals between the two samples.

The internal consistency and test–retest reliability of the subscales was satisfactory to excellent, and the subscales were essentially unrelated to demographic features. Although the internal consistencies were around the traditional cut-off of 0.70, changing to a 4-point scale is hoped to further improve the internal consistence of the scales, as a 3-point scale scoring method may suppress internal consistency because of the limitation in the range of scores, particularly in non-clinical samples.

Both the RFQ_U and RFQ_C scales discriminated between patients and controls, although the RFQ_U scale was clearly superior in this respect as it was more strongly related to BPD diagnosis compared to the RFQ_C. This finding is congruent with other studies finding that BPD patients have significant impairments in RF on measures tapping into internally based mentalizing [25].

The RFQ_C scale was associated with comorbidity between BPD and ED diagnosis, which is congruent with other studies suggesting substantial hyper-reflectivity in patients with these features [26, 39, 60]. The RFQ_C was also significantly positively correlated with measures of mindfulness and perspective-taking, supporting its construct validity, and was significantly negatively associated with both self-reported and clinician-reported BPD features, supporting its predictive validity. The RFQ_U was negatively related to other measures of internally based mentalizing, such as mindfulness and perspective-taking. The RFQ_U was also highly significantly related with BPD features, but also with eating disorder features, severity of depression, and impulsivity. Taken together, these findings suggest that the RFQ_U may be a good marker of typical features associated with BPD, although further prospective research is needed to substantiate these conclusions. Further, although most of the correlations between both RFQ scales and related measures of internally based mentalizing were in the expected direction, they were relatively modest in size, suggesting that the RFQ taps into different features as these measures. Interestingly, both scales were basically unrelated to a measure of externally based mentalizing. This is congruent with findings suggesting that internally based and externally based mentalizing are different capacities that are subserved by relatively different neural circuits [20].

Overall, findings from this study are congruent with a recent study with the French translation of the RFQ, reporting theoretically expected relationships between the RFQ subscales and related measures of internally based and externally based mentalizing in both a clinical and a non-clinical sample [42]. Furthermore, another recent study in a sample of female adult BPD patients and healthy controls reported that both RFQ scales differentiated between clinical and non-clinical samples [61]. Hence, taken together, these three studies provide preliminary support for the construct validity of the RFQ.

The findings of this study need to be interpreted in the context of some key limitations. First, the patient sample was somewhat heterogeneous, with substantial comorbidity between the ED and BPD samples. While the mentalization-based approach assumes that impairments in mentalizing are likely to characterize all forms of severe psychopathology, further studies in more homogenous patient samples are needed to clarify the respective roles of the two RFQ subscales. Second, although clinician-rated instruments were included in this study, the majority of the measures used were self-report questionnaires. Third, there is some discussion as to whether Maximum Likelihood (ML) estimation on Pearson correlations or Weighted Least Squares and Variance Adjusted (WLSMV) estimation using polychoric correlations is the most appropriate approach to CFA for Likert-type scales. Several simulation studies [62–64] suggest advantages and disadvantages of both methods. For instance, WLSMV may be less biased and more accurate than ML in estimating the factor loadings, but may overestimate correlations among factors, particularly when sample sizes are small (*N* < 200) and data are not normally distributed (two conditions that are typical in much of research in clinical psychology, as in the present study). Furthermore, ML may be superior in terms of handling missing values, but on the whole differences between both methods are typically small, and using Pearson correlations or polychoric correlations also seems to lead to comparable results. Holgado et al. (2010, p. 165), for instance, found that values of global indices of goodness of fit, such as the Global Fit Index, the Adjusted Global Fit Index and RMSEA, were generally good enough in both cases (Pearson and polychoric) and led to similar conclusions. Yet, future studies may do well to compare both estimation approaches (ML and Pearson correlations versus WLMSV with polychoric correlations). Finally, further studies are needed to investigate the relationship between the RFQ and experimental and narrative-based measures of mentalizing, an issue we will address in Study 3 in this paper.

Despite these limitations, together with two recent similar studies with the French version of the RFQ [42, 61], this study lends preliminary evidence for the reliability and validity of the RFQ as a brief measure of RF.

## Study 2: Psychometric features of the RFQ in personality disordered patients and normal controls: Replication and extension

The aim of Study 2 was twofold. First, we aimed to replicate the factor structure of the RFQ in a heterogeneous sample of carefully screened patients with personality disorders and in a large non-clinical sample. Second, we wanted to replicate and extend findings concerning the relationships between the RFQ subscales and indices of clinical functioning in patients with personality pathology.

We hypothesized that a two-factor structure consisting of RFQ_C and RFQ_U would provide the best fit to the data. Further, we expected both RFQ_U and RFQ_C to be associated with clinical status, but wished to see if both scales were necessary to achieve this discrimination, given the overlap between the scales in the clinical group. Finally, we expected both subscales to be associated with BPD diagnosis as assessed with the Structured Clinical Interview for DSM-IV Disorders (SCID) in the clinical sample but, in line with our theoretical framework for BPD and findings from Study 1, we anticipated that in an adult population RFQ_U would account for most of the discrimination. We also expected both subscales to be associated with core features of personality pathology, that is, self-harm, personality organization, severity of depression, problems with the regulation of anger, symptomatic distress, interpersonal problems, and decreased well-being.

### Methods

#### Participants and procedures

Participants in the clinical sample were drawn from a sample of 150 patients consecutively admitted to a specialized treatment program for personality disorders at the University Psychiatric Center, Leuven, Belgium, between May 2008 and June 2010, who were asked to participate in a naturalistic outcome study. Patients were referred for treatment by secondary and tertiary community mental health institutes. Inclusion criteria were: (a) primary diagnosis of personality disorder according to DSM-IV, (b) age between 18 and 60 years, and (c) Dutch literacy. Exclusion criteria were kept at a minimum to ensure maximal representativeness of daily clinical practice, and were restricted to: (a) psychotic disorders (except short, reactive psychotic episodes), (b) antisocial personality disorder, (c) severe addictions, and (d) psychiatric disorders secondary to medical conditions.

All 150 patients were asked to participate; 17 patients either left the hospital during the first few days or failed to fill in any questionnaires and four patients did not agree to participate, resulting in 129 patients who were included in the final analyses. After obtaining written informed consent, patients (75% female, mean age 29 years) were enrolled in the study. Most of them were living alone (30%) or living at their parents’ home (42%), and only 22% of them were living together or married. All patients had at least one personality disorder (see Table 6) as assessed with the Structural Clinical Interview for DSM-IV Axis II Disorders (SCID-II) [65]. Overall, 61% of patients were diagnosed with BPD. All SCID interviews were conducted by clinical psychologists and psychiatrists who followed a 2-day training after which they had to pass a reliability test. During the study, they were supervised by an experienced interviewer.

**Table 6:** Prevalence of personality disorders in the clinical sample.

Axis II disorders	N (%)
*Cluster A*	
Paranoid	29 (27%)
Schizoid	4 (4%)
Schizotypal	5 (4.6%)
*Cluster B*	
Borderline	66 (61%)
Antisocial	0
Histrionic	2 (1.8%)
Narcissistic	12 (11%)
*Cluster C*	
Dependent	15 (14%)
Avoidant	43 (39%)
Obsessive-compulsive	31(20.7%)
Personality disorder not otherwise specified	9 (8%)

Participants in the non-clinical sample were drawn from the first wave of a prospective study of heterosexual, biological first-time parents of a healthy child between the ages of 8 and 13 months. During a first home visit, written informed consent was obtained and participants were requested to complete a series of questionnaires, including the RFQ (see below). During a second home visit, which took place approximately 2 weeks later, participants returned the completed questionnaire booklets and were interviewed (interview data not reported in this paper). Of the 294 consenting participants, 287 returned their questionnaire booklet (response rate: 97.6%). Of these 287, six participants were excluded due to too many missing values (i.e., more than 5% missing data), resulting in a final sample of 281 participants (95.6%), consisting of 140 men (49.8%) and 141 women (50.2%). The majority of participants (91.4% of men and 92.9% of women) had Belgian nationality. Men were on average significantly older than women (*t* = 4.73, *p*<0.001), with men averaging 31.18 years of age (*SD* = 4.44) and women 28.99 (*SD* = 3.12). On average, participants reported having been in a relationship with their current partner for approximately 8 years (*SD* = 3.34), and to have been living together for approximately 5 years (*SD* = 2.35). Most couples were married (61.6%) and had been so for almost 3.5 years (*SD* = 2.61). Most participants (70%) held a university degree and were employed (85%) at the time of the study.

This Study was approved by the University of Leuven Social and Societal Ethics Committee.

#### Measures

Participants in both the clinical and non-clinical samples completed the RFQ as part of a larger assessment battery. In addition, in the clinical sample, patients also completed the following assessment instruments:

The Structural Clinical Interview for DSM-IV Axis I (SCID-I) and Axis II disorders (SCID-II) [65, 66]. All patients underwent a standard assessment of personality pathology at intake, including a semi-structured interview to measure Axis I and Axis II disorders. Both interviewers were experienced psychologists who were trained in the Dutch version of the SCID-I and SCID-II.

Patients also completed a list of other measures. The Self-Harm Inventory (SHI) [67] is a 22-item self-report scale used to assess the extent to which patients report engaging in self-injurious behavior [68].

The Inventory of Personality Organization (IPO) [69] is a 136-item self-report measure. All items have a 5-point Likert-type format (1 = never true, 5 = always true). The three main scales (identity diffusion, primitive psychological defense mechanisms, and problems with reality testing) were used in this study. Research has shown excellent internal consistency and test–retest reliability, as well as support for the convergent, concurrent and discriminant validity of the IPO [69].

The Diagnostic Inventory for Depression (DID) [70] is a 38-item self-report scale that assesses both symptom frequency and severity of depression based on DSM-IV criteria, as well as psychosocial impairment and quality of life. The DID has good convergent and discriminant validity, as well as high levels of test–retest reliability [70].

The State-Trait Anger Inventory (STAXI) [71] assesses state and trait anger. In addition, the measure assesses three anger regulation strategies: turning anger towards the self (anger-in), towards others (anger-out), and the control of anger (anger-control).

The Short List of Complaints (SLC) [72] is a self-report questionnaire consisting of 13 questions, scored on a 5-point Likert scale, ranging from 0 (no complaints) to 4 (many complaints), assessing the most frequent psychic complaints or symptoms such as anxiety, depressive feelings, and sleep problems. The total score is calculated with a minimal score of 0 and a maximum score of 52.

The Inventory of Interpersonal Problems-Circumplex (IIP) [73] was used to assess interpersonal problems. The IIP is a self-report measure that includes items assessing interpersonal behaviors that respondents identify as “hard to do” or “does too much” on a 0 (not at all) to 4 (extremely) Likert-type scale. In this study, the average total score of all 64 items was used as a measure of the general level of interpersonal problems.

The Amsterdam Scale of Well-Being (ASWB) [74] assesses well-being rated on the six dimensions described by Ryff (1989). In this study, only the six subscales (39 items) as originally described and validated by Ryff (e.g., [75]) were included, that is, positive relations, autonomy, self-acceptance, environmental mastery, purpose in life, and personal growth. All items are scored on 6-point Likert scale, ranging from 1 (“I don’t agree”) to 6 (“I totally agree”). In this study, we used a total well-being scale calculated as the sum of the six subscales.

#### Statistical analyses

As in Study 1, the factor structure of the RFQ was investigated using (multi-group) CFA with maximum likelihood estimation using the maximum likelihood method in AMOS (Version 4.01) [53], using similar fit indices: a model in which χ^2^/df was ≤3, the CFI and NNFI values were greater than 0.90, and the RMSEA index was between .00 and .06 with confidence intervals between 0.00 and 0.08 [54] was considered acceptable. As in Study 1, we limited the number of possible error correlations to a minimum, allowing only error correlations between items that were similar in formulation or meaning. Multi-group CFA compared a fully unconstrained model (Model 1) with four other models as described in Study 1. In order to compare these five models, *χ*^*2*^-difference tests were used.

Simple *t*-tests and binary regression analyses were used to investigate the ability of the RFQ to discriminate between BPD patients and normal controls. Relationships of the RFQ with clinical features were assessed by computing Pearson correlations.

### Results

#### Confirmatory factor analyses

The RFQ_C and RFQ_U items were subjected to a CFA in the clinical and non-clinical sample separately. The initial models did not provide a good fit to the data, χ^2^/df = 3.25; RMSEA = 0.09 (95% CI = 0.08–0.11); CFI = 0.81, NNFI = 0.76 and χ^2^/df = 2.66 in the non-clinical sample, and RMSEA = 0.12 (95% CI = 0.09–0.14); CFI = 0.78, NNFI = 0.73 in the clinical sample. Modification indices suggested adding error covariances between several items with similar wording and/or content, which resulted in a model with a good fit: χ^2^/df = 1.76; RMSEA = 0.05 (CI = 0.03–0.07); CFI = 0.94, NNFI = 0.92, and χ^2^/df = 1.86 in the non-clinical sample, and RMSEA = 0.07 (CI = 0.04–0.10); CFI = 0.93, NNFI = 0.90 in the clinical sample. Multi-group CFA tested the invariance of the final model obtained in Study 1 across both groups. This model immediately showed a good fit, with the fully unconstrained model showing the best fit, χ^2^/df = 1.59; RMSEA = 0.04 (CI = 0.03–0.05); CFI = 0.95, NNFI = 0.92, suggesting factorial invariance. Other models were associated with a significantly worse fit (measurement weights model: Δχ^2^ = 174.207, *p*<0.01; measurement intercepts model Δχ^2^ = 650.328, *p*<0.001; structural covariances model Δχ^2^ = 656.634, *p*<0.001; measurement residuals model Δχ^2^ = 1485.418, *p*<0.001). Internal consistencies were 0.73/0.78 for RFQ_C and 0.77/0.54 for RFQ_U, respectively. These are satisfactory, again with the exception of the RFQ_U scale in the non-clinical sample, which might again be due to the low average scores on this subscale and thus restriction of range in scores in this community sample.

#### Group differences

Table 7 displays the mean scores for the clinical and non-clinical samples. Both the RFQ_C and the RFQ_U differentiated between clinical and non-clinical participants (*t* = 5.98, *p*<0.001, and *t* = -14.61, *p*<0.001, respectively). When entered simultaneously in a binary regression analysis, the RFQ_C was not associated with a higher odds of belonging to the non-clinical group (OR = 1.05, CI = 0.92–1.20, n.s.), whereas the RFQ_U was highly significantly associated with clinical status (OR = 2.91, CI = 2.30–3.67, p <.01).

**Table 7:** Group differences between the clinical and non-clinical samples.

	Mean	SD
RFQ_C		
Non-clinical	3.1611	2.70487
Clinical	1.6198	2.17277
RFQ_U		
Non-clinical	0.4708	0.97407
Clinical	4.6033	3.04269

Furthermore, the RFQ_U (OR = 1.31, CI = 1.12–1.53), but not the RFQ_C (OR = 0.94, CI = 0.77–1.16), was associated with BPD diagnosis in the clinical sample as assessed with the SCID-II interview in a binary regression analysis with both RFQ scales entered simultaneously.

#### Relationships with clinical features

Table 8 displays the correlation of the RFQ subscales and clinical features in the clinical sample. It shows that the RFQ_U was highly significantly related with core features typically associated with personality disorders and BPD in particular. The RFQ_U was associated with increased levels of self-harm, indices of maladaptive personality functioning as assessed with the IPO, higher levels of depression, greater social impairment, and lower quality of life as assessed with the DID. The RFQ_U was also associated with difficulties with anger regulation, typical of patients with personality pathology, as expressed in higher levels of both state and trait anger, turning of anger towards the self and others, and particularly with problems with anger control, a key feature of patients with borderline levels of functioning. The RFQ_U was also associated with high levels of symptomatic distress, interpersonal problems, and low well-being.

**Table 8:** Relationship between the RFQ subscales and clinical features in the clinical sample.

Measure	Clinical features	RFQ_C	RFQ_U
SHI	Self-harm	-0.17	0.33**
IPO	Primitive defense mechanisms	-0.36**	0.52**
	Identity diffusion	-0.41**	0.57**
	Impairments in reality testing	-0.24**	0.54**
	Total	-0.40**	0.59**
DID	Severity of depression	-0.09	0.40**
	Psychosocial impairment	-0.13	0.36**
	Quality of life	-0.08	-0.26**
STAXI	State anger	-0.16*	.035**
	Trait anger	-0.36**	0.37**
	Anger in	-0.13	0.28*
	Anger out	-0.20*	0.17*
	Anger control	0.32**	-0.36**
SCL	Symptomatic distress	-0.17*	0.45**
IIP	Interpersonal problems	-0.16*	0.32**
ASWB	Well-being	0.21*	-0.41**

Note:

*p<0.05,

**p<0.01

The pattern of associations for the RFQ_C looked quite different and was far from the reverse of the pattern of associations observed with the RFQ_U. Correlations with core indices of psychopathology were typically lower than for the RFQ_U. Notably, the RFQ_C was negatively associated with indices of maladaptive personality functioning as measured with the IPO and the primitive defense mechanisms and identity diffusion subscale in particular. The RFQ_C subscale was also negatively related with trait anger and positively related with anger control.

### Discussion and conclusions

Study 2 provides further support for the reliability and validity of the RFQ. The two-factor structure identified in Study 1 was replicated in an independent clinical and non-clinical sample, which provides further evidence for the robustness of the factors. The RFQ_U subscale in particular differentiated between clinical and non-clinical participants, and was associated with BPD diagnosis as assessed with the SCID structured interview. Additionally, the RFQ_U was highly significantly associated with clinical features that have been proposed as core features of personality pathology by many authors from various theoretical orientations, including self-harm [76], indices of structural personality pathology, including identity diffusion, the use of primitive defense mechanisms and impairments in reality testing [77], feelings of depression and increased symptomatic distress [33, 78], interpersonal problems [25, 79, 80], and decreased quality of life and well-being [81, 82].

The pattern of associations for the RFQ_C looked quite different, suggesting that this scale taps into different features of mentalizing from the RFQ_U. Correlations with core indices of psychopathology were typically lower than for RFQ_U. Of note, the RFQ_C was negatively associated with indices of maladaptive personality functioning as measured with the IPO and the primitive defense mechanisms and identity diffusion subscale in particular, consistent with the assumption that a genuine mentalizing stance is associated with a sense of autonomy, agency, and freedom to explore mental states. The RFQ_C subscale was also negatively related with trait anger and positively related with anger control, in line with the assumption that genuine mentalizing is positively associated with affect regulation and effortful control.

## Study 3: The relationship between the RFQ, parental reflective functioning and infant attachment status

The aim of Study 3 was to test one of the key assumptions of the mentalizing approach using the RFQ—that the capacity for RF in the parent is related to infant attachment status through the capacity for parental RF (PRF), that is, the parents’ capacity to see their child as being motivated by intentional mental states [38, 83]. A family climate characterized by a genuine interest and curiosity in mental states in the infant is expected to foster the development of a secure base experience, the essential background to secure attachment [84]. Furthermore, caregivers with infants who are securely attached to them are expected to “know” their infants’ mind quite well, but part of this knowledge is recognizing that they can never be fully certain about their child’s mental states, thus recognizing the mind’s opacity [23].

Although one study found that parents’ general level of RF as assessed on the AAI was related to infant attachment status [85], it is typically assumed that the capacity of the parent to reflect on the mental states of their infant in particular, that is, PRF, is more closely related to infant attachment status. Indeed, it is not the parents’ general capacity for mentalizing, but their capacity in relation to a particular infant that is expected to influence the development of that infant’s attachment security. Although general RF and PRF can be expected to be correlated, clearly, both capacities need not be identical. The correlation of attachment classification between twins is moderated by the specific sensitivity the parent shows to each sibling [86]. Consistent with this assumption, a study by Steele et al. [87] found a good but not perfect correlation (*r* = 0.50) between general RF as scored on the AAI and PRF as scored on the Parent Development Interview [88].

Most studies in this area have thus focused on the relationship between PRF specifically and infant attachment status [83, 89–93]. Very few studies have simultaneously investigated the relationship between general RF and PRF and infant attachment status [89]. In this study, we hypothesized that general RF as assessed with the RFQ would be related to features of PRF as assessed with the Parental Reflective Functioning Questionnaire, which in turn were expected to be related to infant attachment security. Hence, we expected PRF to mediate the relationship between RF and infant attachment security as assessed with the Strange Situation Procedure (SSP), an experimental procedure involving brief separation and reunion episodes with a primary caregiver [84].

### Methods

#### Participants and procedures

Participants were parents and their infants that were assessed at 10 months of age at the Anna Freud Centre, London, UK, as part of a broader study on parent–infant relationships. During this visit, after written informed consent, parents completed a series of questionnaires and experimental tasks related to parenting attitudes. Parents and infants returned 2 months later to a different part of the laboratory, at which time the infant’s attachment security was assessed using the SSP.

The sample for this study was drawn from a database of parents from north-west London who agreed to be contacted regarding developmental studies. In total, 224 mothers and infants (46.8% boys) were recruited. As the RFQ and Parental Reflective Functioning Questionnaire (PRFQ; see below) were added to the battery at a later date, only 136 parents and their infants could be included in the current study. There were no differences, however, in any of the demographic features between mothers in the original sample and those who also completed the PRFQ. Demographic features of mothers and infants in Study 3 are summarized in Table 9. Most mothers were Caucasian and had attained higher education, and about half of the infants were female.

**Table 9:** Demographic features of participants.

Mean maternal age in years (SD)	34.24 (3.58)
Annual household income % <£30,000	19.1
Marital status, % married or living with partner	89.7
Education	
% Secondary education	10.3
% Further education	16.9
% Higher education	72.8
Mother ethnicity	
% Caucasian	88.1
% Other	11.6
Baby gender	
% (n) Male	46.3 (63)
% (n) Female	53.7 (73)

Study 3 has been approved by University College London Research Ethics Committee.

#### Measures

The SSP consists of eight 3-minute episodes during which the mother leaves (separation episodes) and rejoins (reunion episodes) the infant twice. All SSPs were videotaped and coding was done by a trained coder who had passed reliability assessments on 48 tapes (kappa = 0.78 on two- way, 0.73 for three-way attachment classifications and 0.80 for disorganized), using the criteria outlined by Ainsworth [84]. In total, 95 infants were classified as secure and 41 as insecure.

The Parental Reflective Functioning Questionnaire (PRFQ) is a brief measure of PRF, assessing (a) prementalizing modes, (b) certainty about the mental states of the infant, and (c) interest and curiosity in the mental states of the infant. Studies have supported the reliability and validity of these three subscales in that they are related in theoretically predicted ways to parental attachment dimensions, emotional availability, parenting stress, infant attachment status in the SSP [94] and distress tolerance in a simulated baby paradigm [95].

#### Statistical analyses

Pearson correlations between the RFQ subscales, the PRFQ, and infant attachment insecurity as assessed in the SSP were calculated. Binary regression analysis was used to investigate the association between the RFQ subscales and infant attachment security in the SSP. Structural equation modeling was used to examine the proposed mediation model using AMOS [53]. In a first step, we tested a base model with all the paths included (hence including both direct and indirect paths from the RFQ to infant attachment security). Non-significant paths were deleted, which yielded the final base model. The goodness of fit of the models tested was evaluated using several fit indices discussed in Studies 1 and 2: the *χ*^*2*^-index, RMSEA, NNFI, and CFI.

### Results

Table 10 shows that the RFQ_C, but not the RFQ_U, subscale was correlated with infant attachment insecurity. Further, the RFQ_C subscale was positively correlated with the Certainty of Mental States scale from the PRFQ, providing support for its convergent validity. The RFQ_C subscale was negatively associated with the PRFQ Prementalizing subscale.

**Table 10:** Zero-order correlations between the RFQ, PRFQ, and infant attachment security as assessed in the SSP.

	2	3	4	5	6
1. Attachment Insecurity	-0.162*	0.106	0.265**	-0.047	-0.225**
2. RFQ_C	–	-0.349**	-0.291*	0.413**	0.024
3. RFQ_U		–	0.287*	-0.268*	0.013
4. PRFQ Pre-Mentalizing Modes			–	-0.108	-0.201*
5. PRFQ Certainty of Mental States				–	-0.093
6. PRFQ Interest and Curiosity in Mental States					–

Note:

*p<0.04,

**p<0.01.

The RFQ_U subscale, in turn, was positively correlated with the PRFQ Prementalizing subscale and negatively correlated with the PRFQ Certainty About Mental States subscale.

Binary regression analyses showed that the RFQ_C predicted infant attachment security, OR = 1.30 (*p*<0.05), whereas there was almost no trend for the RFQ_U scale to predict infant attachment insecurity (*p* = 0.19).

**Fig 2:**
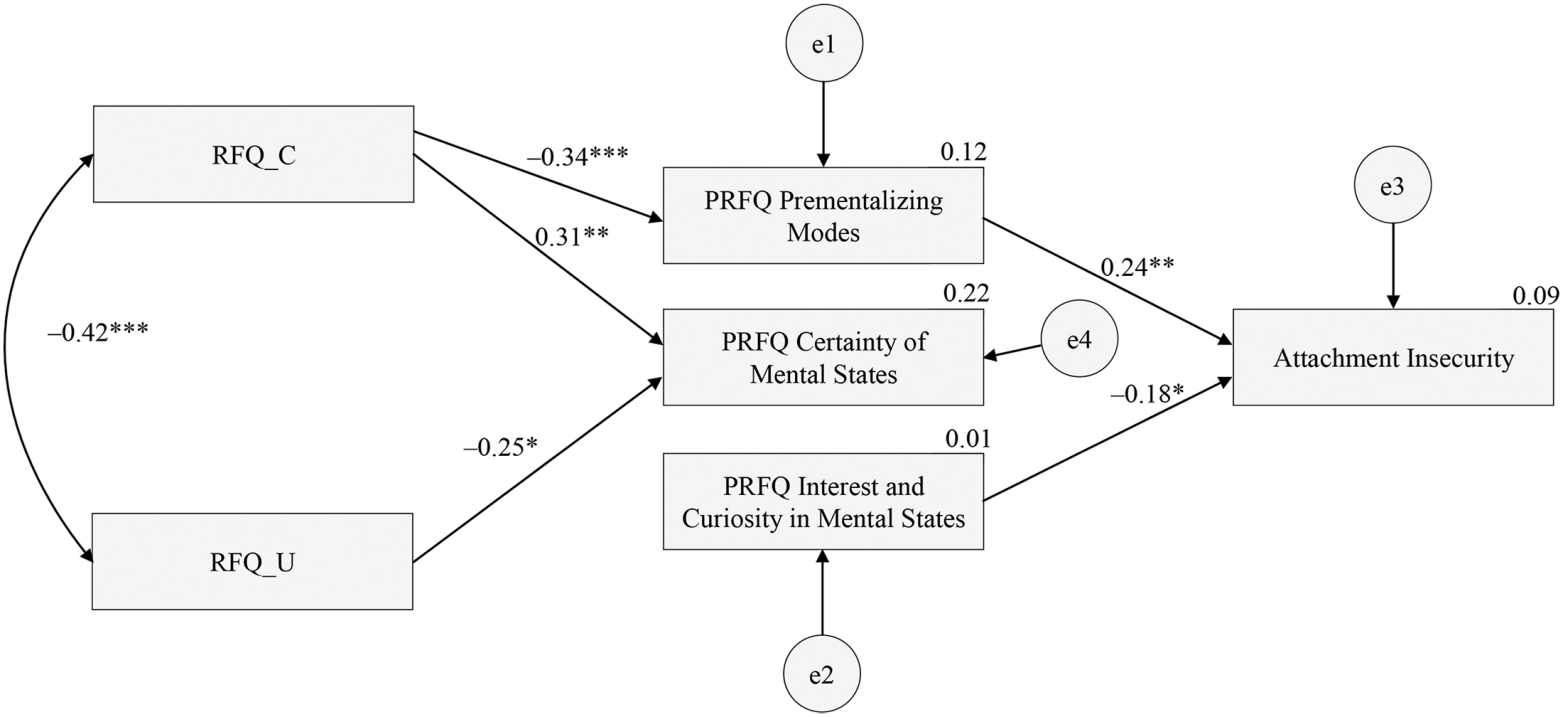
Final model for the relationships between the RFQ, PRFQ, and infant attachment status as assessed in the SSP. Rectangles indicate measured variables and the small circles reflect residuals (e). Bidirectional arrow depicts covariance and unidirectional arrows depict hypothesized directional links. *p<0.05, **p<0.01, ***p<0.001.

Structural equation modeling showed that the initial theoretical model did not provide a good fit to the data. Deleting non-significant paths led to excellent model fit, χ^2^/df = 0.16, CFI = 0.98, NNFI = 0.94, RMSEA = 0.03 (95% CI = 0.00–0.08). As Fig 2 shows, the RFQ_C subscale was negatively related to the PRFQ Prementalizing subscale, which in turn was related to infant attachment insecurity. As the direct path from RFQ_C to infant attachment security was not significant, this provides evidence for an indirect effect of RFQ_C on attachment security through mothers’ tendency to think in biased and negative ways about their infant’s mental states. RFQ_C was also related to PRFQ Certainty about Mental States. Interestingly, RFQ_U was not related to infant attachment security, but was negatively related to PRFQ Certainty about Mental States. Finally, PRFQ Interest and Curiosity was not related to the RFQ but did predict infant attachment security, suggesting a second pathway to infant attachment security.

### Discussion

The results of this study provide evidence the RFQ_C was related to infant attachment insecurity. Mediation analyses suggested that this effect was explained by the negative association of RFQ_C with the absence of maladaptive attributions concerning one’s child. This finding is congruent with the assumption that high levels of RF are associated with a relative absence of such attributions [38]. The association was as predicted and is hardly counterintuitive. It is very hard to imagine how poor RF and high levels of prementalizing modes of thinking in relation to parenting could lead to secure attachment. Prementalizing modes of experiencing subjectivity on the part of caregivers are typically expressed in a tendency to make malevolent attributions and an inability to enter into the child’s internal subjective world, features that studies suggest are characteristic of parents with severe mentalizing problems [96–100]. Recognizing the opacity of mental states and showing a genuine interest and curiosity in the internal world of the child are considered to be the hallmarks of genuine PRF [101], and these two features were indeed associated with RFQ_C in the current study.

Findings from this study are also congruent with a study reporting only a modest correlation between general RF as assessed on the AAI and PRF as rated on the Parent Development Interview [87]. In this study *r*s ranged from non-significant to *r* = 0.35, depending on the specific features of PRF, further suggesting that both capacities show only modest overlap. Another study reported modest correlations between general RF and RF with regard to traumatic experiences [102], again pointing to the domain-specific character of reflective capacities. In line with this assumption, the current findings suggest that general RF and PRF are two relatively distinct capacities, probably loosely coupled by certain shared processing elements.

The RFQ_U subscale, while clearly related to indices of psychopathology in both Study 1 and 2, was not related to infant attachment security. RFQ_U was only negatively related with PRFQ Certainty about Mental States. The absence of a strong association between this subscale and features of PRF and infant attachment status in this study might well be explained by the relatively high-functioning characteristics of this sample, with few if any participants likely to meet clinical caseness criteria. In a clinical sample, the level of RFQ_U may be more predictive of insecure and particularly disorganized infant attachment status. Future research is needed to address this issue.

Findings from Study 3 need to be interpreted with caution. First, this study was cross-sectional, and causal inferences are thus speculative. Second, the proportion of infants classified as disorganised was small in this low-risk sample and the association of RFQ_U with this attachment category could not be explored. Further research with high-risk samples is needed. Similarly, limited statistical power and the skewedness of the sample towards secure attachment precluded investigating the association between the RFQ subscales and specific insecure attachment classifications.

## General discussion and future perspectives

These three studies together provide preliminary evidence for the reliability and validity of the RFQ. The RFQ shows satisfactory reliability and test–retest reliability, although reliability of the RFQ_U subscale can be an issue in normal community samples due to limited spread of scores. It is hoped that a 4-point scale will improve on this limitation of the instrument. The results furthermore suggest that the RFQ_U is superior to the RFQ_C subscale in predicting clinical caseness and BPD diagnosis in particular, as well as key features of personality pathology. RFQ_C, in turn, was generally negatively related to caseness, but may be more specifically related to types of psychopathology characterized by hypermentalizing, as is, for instance, observed in patients with eating disorders [13, 60, 103] or adolescents with BPD features, [104, 105]. For instance, a recent study showed that the RFQ_C scale was associated with non-suicidal self-injury [42]. Furthermore, the RFQ_C, but not RFQ_U, was related to infant attachment security, providing further evidence for their distinction. Although there is overlap in the items used to calculate both RFQ subscales, consistent with theoretical assumptions, findings of this study suggest that they are tapping into different processes. The subscales have different relationships with indices of psychopathology, with PRF, and with infant attachment. One hypothesis, requiring further exploration, places excessive certainty about mental states at a higher level of functioning and at an earlier developmental stage in the emergence of personality problems. Given the ubiquity of mentalizing in human culture [106], it is possible to see excessive certainty and the proliferation of hypotheses about the content of mental states in others as an adaptation to a maturational weakness or an emerging underlying deficit in the processing of mental state contents.

Although further research with the RFQ in other samples is needed, findings of the studies reported in this paper provide preliminary evidence for its reliability and validity and highlights possibilities for its broader application. The generic nature of the association to psychopathology reported above indicates that mentalizing problems identified by the RFQ may have a role to play in many (if not all) mental disorders. This assumption is consistent with both factor analytic (e.g., [107]) and neurobiological (e.g., [108, 109]) studies of psychopathology. A series of recent reports has identified a generic factor (variously labeled the “g” or “p” factor) in community-based studies of both adult and pediatric samples [110–112]. We hypothesize that RF or mentalizing plays a central role in creating a general developmental vulnerability to mental disorders, which could be explored using the RFQ. Individuals with lower RFQ scores on either or both factors in longitudinal studies should show greater propensity for psychopathology, with environmental factors and life events most likely serving as moderators and triggers of the expression of specific disorders. Further longitudinal, population-based research is required to explicitly test this hypothesis. The fact that the RFQ consists of only 8 items will facilitate its application to epidemiological studies with large clinical and non-clinical samples. Longitudinal studies may get closer to establishing causal pathways. Further validation also needs to establish whether the RFQ predicts performance in experimental assessments of mentalizing. In addition, further validation using functional imaging techniques with clear predictions of the relative activation of specific neural circuits putatively associated with mentalizing would be of great interest.
